# Making Traditional Japanese Distilled Liquor, Shochu and Awamori, and the Contribution of White and Black *Koji* Fungi

**DOI:** 10.3390/jof7070517

**Published:** 2021-06-28

**Authors:** Kei Hayashi, Yasuhiro Kajiwara, Taiki Futagami, Masatoshi Goto, Hideharu Takashita

**Affiliations:** 1 Sanwa Research Institute, Sanwa Shurui Co., Ltd., Usa 879-0495, Japan; kajiwara-y@kokuzo.co.jp (Y.K.); takashita-h@kokuzo.co.jp (H.T.); 2 Education and Research Center for Fermentation Studies, Faculty of Agriculture, Kagoshima University, Kagoshima 890-0065, Japan; futagami@agri.kagoshima-u.ac.jp; 3 United Graduate School of Agricultural Sciences, Kagoshima University, Kagoshima 890-0065, Japan; mgoto@cc.saga-u.ac.jp; 4 Department of Applied Biochemistry and Food Science, Faculty of Agriculture, Saga University, Saga 840-8502, Japan

**Keywords:** *koji*, shochu, honkaku shochu, awamori, *Aspergillus luchuensis*, Aspergillus luchuensis mut. *Kawachii*, *Aspergillus awamori*, multiple parallel fermentation, amylolytic enzymes system, citric acid synthesis, wide variety of flavors, genome editing technology

## Abstract

The traditional Japanese single distilled liquor, which uses *koji* and yeast with designated ingredients, is called “honkaku shochu.” It is made using local agricultural products and has several types, including barley shochu, sweet potato shochu, rice shochu, and buckwheat shochu. In the case of honkaku shochu, black *koji* fungus (*Aspergillus luchuensis*) or white *koji* fungus (*Aspergillus luchuensis* mut. *kawachii*) is used to (1) saccharify the starch contained in the ingredients, (2) produce citric acid to prevent microbial spoilage, and (3) give the liquor its unique flavor. In order to make delicious shochu, when cultivating *koji* fungus during the shochu production process, we use a unique temperature control method to ensure that these three important elements, which greatly affect the taste of the produced liquor, are balanced without any excess or deficiency. This review describes in detail the production method of honkaku shochu, a distilled spirit unique to Japan and whose market is expected to expand worldwide, with special attention paid to the *koji* fungi cultivation step. Furthermore, we describe the history of the *koji* fungi used today in the production of shochu, and we provide a thorough explanation of the characteristics of each *koji* fungi. We also report the latest research progress on this topic.

## 1. Introduction

Traditional Japanese distilled liquors include shochu and awamori [1]. Shochu is divided into two types, single distilled shochu and continuously distilled shochu, depending on the distillation method used under the Japanese Liquor Tax Law. Among single distilled liquors, those using *koji* and yeast with designated ingredients are specifically called “honkaku shochu”. *Koji* is a fermented grain, such as rice and barley, with filamentous fungi (called *koji* fungi). *Koji* produces saccharification enzymes, which convert the starch contained in the ingredients into mono- or disaccharides which can be further utilized by yeast to produce ethanol [2].

Originally, honkaku shochu was mainly enjoyed in the Kyushu region in the southern part of Japan, but it began to spread throughout Japan around 1980; in recent years, it has also been enjoyed overseas. Honkaku shochu is made using local agricultural products as fermented ingredients. There are various types of honkaku shochu, such as barley shochu, sweet potato shochu, rice shochu, and Japanese buckwheat (soba) shochu. In addition to alcohol, it contains an abundance of other characteristic flavor components. In contrast, awamori is a distilled liquor that has been enjoyed mainly in the Okinawa Prefecture since the Ryukyu Kingdom era. The characteristic of its making method is that rice *koji* produced using black *koji* fungus is used as an ingredient, and fermentation is carried out in a single step [3].

In this review, we discuss the preparation of single distilled shochu, in which *koji* fungi are an important factor, and describe the types and characteristics of *koji* fungi used in the making of shochu.

## 2. Making Method of Single Distilled Shochu

**Figure 1:**
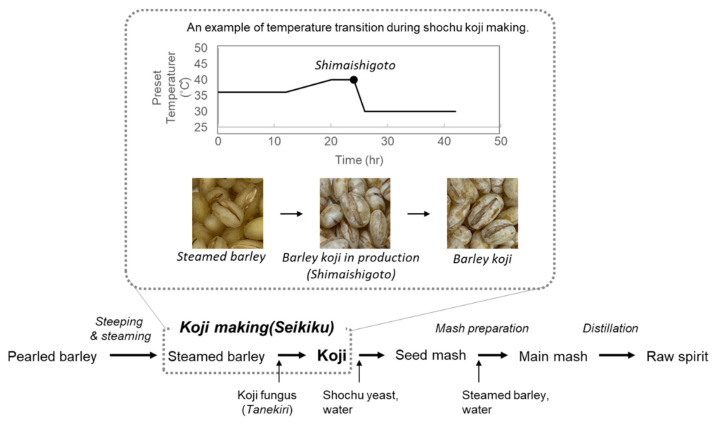
Making method for single distilled barley shochu.

The conventional first step in the making of single distilled shochu, grain ingredients, such as rice and barley, are inoculated with *koji* fungi, and it is fermented in a solid state to make *koji* (Figure 1). The main purpose of using *koji* in the making of liquor is to provide an enzyme source that decomposes starch, protein, and lipids, as well as to supply vitamins that enhance the growth of yeast in mash [4]. In addition, other characteristics of *koji* fungi are crucial for the making of honkaku shochu and awamori. Currently, yellow *koji* fungus is used for making sake, whereas many shochu factories use black and white *koji*, because they can produce large amounts of citric acid. By using *koji* with a high content of citric acid, the pH of the mash can be kept low, and the growth of microorganisms other than the yeast involved in alcoholic fermentation can be suppressed [5]. As a result, a stable and good fermentation becomes possible even in the hot, humid, and harsh climate of Japanese summers or in an open environment.

The second step is alcoholic fermentation. In honkaku shochu, fermentation is generally divided into two stages [6]. Briefly, shochu yeast and water are added to the *koji* produced in the first step to create a starter culture (seed mash). Once the yeast has grown sufficiently in the starter culture, the main ingredients are added to make the main mash, and alcoholic fermentation takes place. During this period, multiple parallel fermentation is performed, in which saccharification of the ingredient by each enzyme derived from the *koji* fungus and fermentation by yeast occur simultaneously.

In multiple sequential fermentation, which separates saccharification and fermentation (e.g., beer and whiskey), the alcohol content at the end of fermentation is approximately 5–8%. In multiple parallel fermentation, saccharification and fermentation occur in a well-balanced manner, and the alcohol content at the end of fermentation can be as high as 15–20%.

The third step is distillation. While whiskey is distilled twice or more, honkaku shochu is distilled once by a single distillation method (pot still) [7]. Because the alcohol content in the mash stage is high, a high concentration of alcohol can be obtained by distillation. A characteristic of this step is that the flavor specific to the main ingredient can be transferred to the distilled liquor.

## 3. *Koji* Fungi Used for Shochu and Awamori

Until the early 1900s, *koji* for shochu making consisted exclusively of *Aspergillus oryzae*, because sake making using this yellow *koji* fungus had already been developed in Japan. *A. oryzae* strains cannot produce high amounts of organic acids. Prior to the start of shochu making, in southern Ryukyu Islands of Okinawa, awamori was being made using the black *koji* fungus, *Aspergillus awamori* or *A. luchuensis,* which can produce large amounts of citric acid. At around 1910, shochu breweries in Kagoshima, Japan, introduced the black *koji* fungi from Okinawa to make *koji* for shochu [8]. This enabled stable saccharification and fermentation during shochu production. In the early-1900s, a white colony was isolated from black colonies of *A. awamori* in order to obtain colorless mutants [9]. The resultant albino mutant strain was termed white *koji* fungus, *A. kawachii*, which maintains the amylolytic activity and productivity of citric acid in *koji* [10]. The three *koji* fungi used for producing shochu or awamori belong to *Aspergillus* section *Nigri*, which includes *koji* fungi and *A. niger*. These have morphologically similar phenotypes; however, some strains of *A. niger* produce ochratoxin and/or fumonisin, whereas the three *koji* fungi cannot produce mycotoxins [11,12]. The taxonomic classification of *Aspergillus* section *Nigri* was reevaluated, because *A. awamori* contains various *Aspergillus* species other than the original, and some of these species were isolated from circumstances other than the place for *awamori* production [13]. Molecular genetic analysis demonstrated that black and white *koji* fungi are taxonomically identical and different from *A. niger*. Therefore, the *A. awamori* used for making shochu and awamori was proposed to be renamed as *A. luchuensis*, and *A. kawachii* as *A. luchuensis* mut. *kawachii*.

## 4. Temperature Control for Shochu *Koji* Making

After inoculation of the *koji* fungus *A. luchuensis* to steamed rice or barley grains (termed *Tanekiri*), the cultivation temperature gradually increases to approximately 40 °C due to the heat generated by the growth of *A. luchuensis*; the temperature is then lowered to approximately 30 °C by a mixing and cooling processes called “*Shimaishigoto*” [14] (Figure 1). This temperature modulation is considered to be developed and refined based on the experiences of shochu craftsman in order to control the production of optimal amounts of amylases and citric acid [14]. The initial stage characterized by higher temperature is known to stimulate amylase activity, whereas the later stage characterized by lower temperature enhances the production of citric acid [15,16].

**Figure 2:**
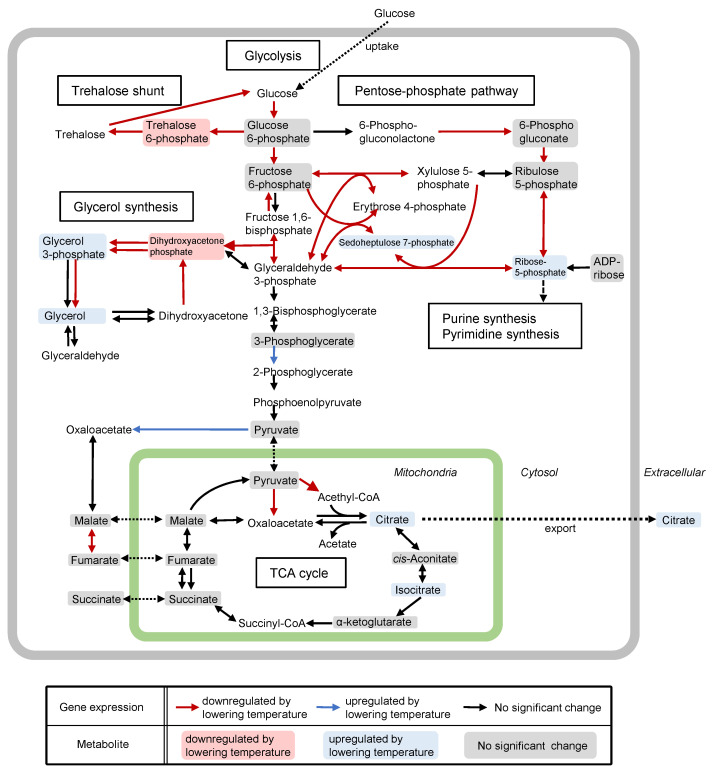
Metabolic map of citric acid synthesis in *Aspergillus luchuensis* mut. *kawachii*. Glycolysis, pentose phosphate pathway, trehalose synthesis pathway, glycerol synthesis pathway, and tricarboxylic acid (TCA) cycle are depicted [17]. Different colored arrows and metabolites indicate upregulated, downregulated, or not significantly changed gene expression and metabolite levels as a consequence of lowering the temperature during *koji* making [17].

Carbon sources such as glucose and sucrose are metabolized to produce pyruvate via the glycolytic pathway; subsequently, citric acid is synthesized by citrate synthase as an intermediate compound of the tricarboxylic acid (TCA) cycle in the mitochondria and excreted into the cytosol prior to subsequent excretion into the extracellular environment (Figure 2) [17,18,19]. Based on the transcriptomic and metabolomic data, the lower temperature causes the downregulation of genes involved in glycerol, trehalose, and pentose phosphate metabolic pathways, indicating that heat adaptation leads to reduced citric acid accumulation through the activation of pathways branching from glycolysis [17]. In addition, significantly reduced expression of genes related to heat shock responses and increased expression of genes related to amino acid transport were also observed after the temperature decrease. Thus, solid-state cultivation at 40 °C is considered to be stressful for *A. luchuensis*, preventing the production of citric acid.

## 5. Amylolytic Enzyme System in *Koji*

**Figure 3:**
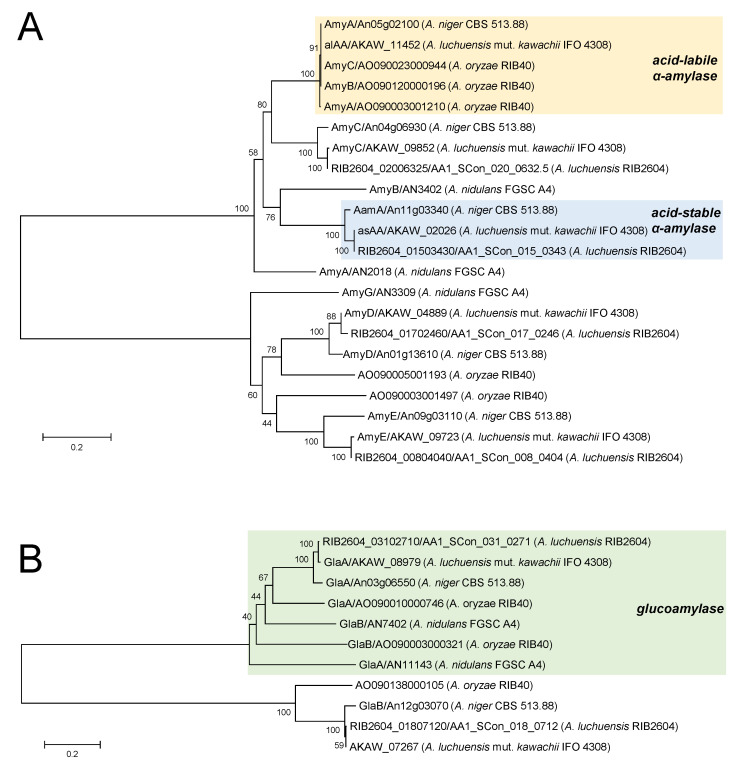
Phylogenetic tree of α-amylases (**A**), glucoamylases (**B**), and some homologs in *Aspergillus oryzae* RIB40, *A. luchuensis* RIB2604, *A. luchuensis* mut. *kawachii* IFO 4308, *A. niger* CBS 513.88, and *A nidulans* FGSC A4. The phylogenetic tree was constructed using the neighbor-joining method based on the alignment of amino acid sequences with pairwise deletion using MEGA v. 6 (http://www.megasoftware.net/ (accessed on 1 May 2021).

The most crucial function of *koji* fungi in the making of sake and shochu is the production of amylolytic enzymes, α-amylase (Amy) (Figure 3A), and glucoamylase (Gla) (Figure 3B). These enzymes play a crucial role in the saccharification of starch to yield glucose and maltooligosaccharides. The maltooligosaccharides formed by the action of Amy are hydrolyzed by α-glucosidase (Agd) in *koji* to form glucose. Agd sometimes transglucosylates glucose as a donor to form isomaltose, ethyl-glucoside, and glucoside compounds. Isomaltose is believed to induce the expression of the genes encoding Amy, Gla, and Agd in the yellow *koji* fungus, *A. oryzae* [20]. In yellow *koji*, AmyB and two glucoamylases (GlaA and GlaB) contribute to the saccharification of starches in rice. AmyB is not tolerant to acidic conditions because of the non-production of large amounts of organic acids by *A. oryzae*. GlaB is characteristically found in *A. oryzae* and is highly expressed under solid-state culture conditions corresponding to those of *koji* making; however, GlaA is highly expressed in liquid culture, but poorly expressed in solid-state culture or *koji*. It is believed that GlaB is adapted to *koji*-making circumstances [21].

The shochu *koji* fungus, *A. luchuensis*, predominantly produces two types of α-amylase, acid-labile α-amylase (alAA) and acid-stable α-amylase (asAA) [22,23] (Figure 3A), and a glucoamylase (GlaA) in *koji* [24] (Figure 3B). AlAA from *A. luchuensis* is closely related to AmyB from *A. oryzae*; however, it is also found in a citric acid producer of *A. niger* but not in *A. oryzae*. GlaA from *A. luchuensis* has a starch binding domain (family CBM_20) at the C-terminus, allowing the digestion of not only gelatinized but also raw starches [25]. This enzyme is closely related to *A. oryzae* GlaA and is phylogenetically separated from *A. oryzae* GlaB [17]. A set of three major amylolytic enzymes, alAA, asAA, and GlaA, from *A. luchuensis* is indispensable for efficient starch saccharification in *koji*. *A. luchuensis* initiates the production of alAA only during the initial period of *koji* making. Then, it initiates the production of asAA and citric acid in *koji*. Deletion of each amylolytic gene results in inefficient saccharification of rice starch and subsequent ethanol fermentation by yeast [26].

Genome analysis of *A. luchuensis* mut. *kawachii* revealed the presence of genes encoding putative amylolytic enzymes, including those that are poorly expressed in *koji*. In addition to alAA and asAA, the putative α-amylases AmyC (AKAW_09852), AmyD (AKAW_04889), and AmyE (AKAW_09723) were found in its genome [17] (Figure 3A). As AmyD and AmyE do not possess N-terminal signal sequences, these putative α-amylases do not appear to be directly involved in the hydrolysis of extracellular substrates. In addition, *amyE* is located in a gene cluster with *agtA* and *agsE*, which are putative 4-α-glucanotransferase and α-1,3-glucan synthase genes, respectively, in *Aspergillus* species, implying that *amyE* is involved in cell wall biogenesis and degradation [27]. In the case of glucoamylase, the *A. luchuensis* genome possesses two, GlaA and GlaB (AKAW_07267) (Figure 3B). Although *A. luchuensis* GlaB has not been well characterized with respect to shochu making, it is known to be phylogenetically distant from *A. oryzae* GlaB, which is crucial for sake making.

## 6. Citric Acid Production in *Koji* Fungi

The genomic and physiological features of *A. luchuensis* are similar to those of *A. niger*, which is industrially used for the production of citric acid [28]. A mathematical model suggested that citric acid overflow might be controlled by the transport process (e.g., uptake of carbon source, pyruvate transport from the cytosol to the mitochondria, transport of citrate from the mitochondria to the cytosol, and extracellular excretion) in *A. niger* [29,30,31]. For example, the gene expression levels of the mitochondrial citrate transporter-encoding *ctpA* and *yhmA* genes were upregulated approximately 1.8-fold by lowering the temperature during the *koji*-making process [18]. This implied that the increased expression level of mitochondrial citrate transporters might have contributed to the increase in citric acid production.

Recently, the plasma membrane-localized citrate exporter, CexA, was identified as a key factor in the accumulation of extracellular citric acid in *A. niger* [32,33]. CexA has also been characterized in the *koji* fungi *A. luchuensis* mut. *kawachii* and *A. oryzae* and has been shown to play a significant role in the accumulation of citric acid in *koji* [19,34]. Disruption of *cexA* causes a significant decline in both extracellular and intracellular citric acid accumulation in *A. luchuensis* mut. *kawachii*, whereas in *A. oryzae*, the overexpression of *A. luchuensis* *cexA* significantly enhances both extracellular and intracellular citric acid accumulation to a level comparable to that of *A. luchuensis*. Interestingly, the *A. oryzae* genome encodes two intrinsic *cexA* homologs, *cexA* and *cexB*. Overexpression of these genes in *A. oryzae* also enhances citric acid accumulation, implying that *A. oryzae* is a potential producer of citric acid.

## 7. Genome Analysis and Editing of *Koji* Fungi

Among *koji* fungi, genome information was first obtained for the yellow *koji* fungus *A. oryzae* RIB40 [35]. Thereafter, genome information became available for *A. luchuensis* mut. *kawachii* IFO 4308 and *A. luchuensis* NBRC 4314 (RIB2604) [17,36,37]. The development of recombinant hosts is also important for understanding shochu *koji* fungi and their applications. To develop a research host for black and white *koji* fungi, a highly efficient homologous recombination strain was constructed by disrupting *ligD* involved in non-homologous end joining (NHEJ) for gene repair in *A. luchuensis* NBRC 4314 and *A. luchuensis* mut. *kawachii* IFO 4308 [38,39]. Furthermore, the development of the clustered regularly interspaced short palindromic repeats (CRISPR)-Cas9 system, a genome editing technology, has allowed gene knockout with high efficiency even in wild strains in which NHEJ functions normally [40]. In the near future, breeding of mutant strains using the genome editing technology, a breakthrough technology for genetic modification, will likely be carried out actively.

## 8. Diversity of Shochu Flavor

Compared to the other white spirits, honkaku shochu has been found to have a milder flavor profile due to its high content of higher alcohols and low boiling esters, as well as low levels of acetic acid, ethyl acetate, and acetaldehyde [41]. Moreover, because of mild and free of miscellaneous flavors, honkaku shochu enables to perceive the variety of flavors that arise from the different regions where it is produced and the different production methods. The following three factors greatly influence its diverse flavors: (i) the main ingredients, such as rice, barley, and sweet potato, used as the fermentation substrate; (ii) selection of the microbial species used; and (iii) the fermentation and distillation methods.

The characteristic flavor components derived from the main ingredients that have been well researched include alkyl furans (2-methylfuran, 2-ethylfuran, and 2-pentylfuran) in barley-based shochu [42] and terpenes (linarol, α-terpineol, citronellol, and geraniol), rose oxide, methyl benzoate, β-damasenone, i-eugenol, β-ionone, and ethyl cinnamate in shochu made from sweet potato [43,44]. In the case of shochu made from buckwheat, ethyl cinnamate was found to be one of the characteristic flavors, although it was also found in sweet potato shochu and rice shochu [45].

Research on how shochu *koji* fungi affect the flavor of honkaku shochu has progressed significantly in the recent years. *A. luchuensis* produces 1-octen-3-ol, an unsaturated alcohol with a mushroom-like flavor [46,47]. 1-octen-3-ol is produced as a by-product during the biosynthesis of oxylipin, which is an oxidized fatty acid involved in the regulation of fungal development. The production of oxylipin and 1-octen-3-ol is mediated by the fatty acid oxygenase Ppo (psi-produced oxygenase). The *ppo* genes have been characterized in *A. luchuensis*, demonstrating that PpoC plays a significant role in the production of 1-octen-3-ol during the *koji* making process. *A. luchuensis* is also involved in the production of vanillin, which is one of the characteristic flavors in aged and mature awamori and shochu. *A. luchuensis* produces ferulic acid (FA) esterase to cleave FA from FA-conjugated hemicellulose in rice grains. The liberated FA is converted to 4-VG by the phenolic acid decarboxylase (Pad) of *A. luchuensis* [48,49]. During distillation, 4-VG is further transformed by abiotic oxidization to yield vanillin. *A. luchuensis* plays a key role in 4-VG production during the awamori making process [50].

In addition, research on yeast, microbial species that affects the flavor of honkaku shochu, is underway. Phylogenetic analysis of *S. cerevisiae* strains used for the making of shochu and awamori showed that they are phylogenetically close to sake yeast strains, but are still distinct groups [51]. Thus, a comparative genomic study of shochu and sake yeasts might enhance our understanding of the genetic features of shochu yeast. Currently, genome information of the shochu yeasts *S. cerevisiae* Kagoshima no. 2 and BAW-6 is available [52,53].

In the process of shochu making, differences in distillation methods are a major factor affecting its flavor. The method of distilling shochu is roughly divided into distilling under atmospheric pressure (atmospheric distillation) and distilling by reducing the pressure in the distillation pot using a vacuum distillation device (vacuum distillation). Shochu produced by atmospheric distillation has a complex flavor as a result of the thermo-chemical reaction of various components contained in the mash, such as, for example, furfural produced from xylose under the acidity of citric acid [54]. In contrast, shochu produced by vacuum distillation has a fruity flavor peculiar to mash. It is also possible to diversify the flavor of shochu by making good use of both distillation methods.

In recent years, the profiling of shochu flavors has been vigorously pursued, with reports on the evaluation and classification of sensory characteristics of ingredients in barley-based honkaku shochu [55], and the creation of flavor wheels for the flavor components of awamori [56].

## 9. Conclusions

Various factors determine the flavor of shochu: (i) the main ingredients used as the fermentation substrate; (ii) the microbial species used; and (iii) the fermentation and distillation methods. In the future, shochu craftsmen may be able to modulate one of these three factors or their combination to produce shochu with characteristic flavors. Moreover, the flavor may be further diversified by combining these factors.

Honkaku shochu and awamori, which has historically been enjoyed in only one area of Japan, have spread worldwide owing to their unique and profound cultural characteristics that make use of the blessings of nature unique to Japan. Both of these liquors have interesting aspects related to maintaining the traditional making method and continuing various innovative technological developments in engineering and microbiology. Honkaku shochu and awamori have the potential to expand their market to a level comparable to that of distilled liquors such as whiskey, brandy, vodka, rum, gin, and tequila.

## Data Availability

Not applicable.
